# Inflammation predicts accelerated brachial arterial wall changes in patients with recent-onset rheumatoid arthritis

**DOI:** 10.1186/ar2668

**Published:** 2009-04-06

**Authors:** Suad Hannawi, Thomas H Marwick, Ranjeny Thomas

**Affiliations:** 1 Diamantina Institute, University of Queensland, Princess Alexandra Hospital, Ipswich Road, Woolloongabba, Queensland 4102, Australia; 2 Department of Medicine, University of Queensland, Princess Alexandra Hospital, Ipswich Road, Woolloongabba, Queensland 4102, Australia

## Abstract

**Introduction:**

Patients with recent-onset rheumatoid arthritis (RA) have impaired brachial artery endothelial function compared with controls matched for age, sex and cardiovascular risk factors. The present study examined endothelium-dependent (flow-mediated dilatation (FMD)) and independent (glyceryl trinitrate (GTN)-mediated dilatation (GMD)) structural responses in early RA patients, and determined progress over one year.

**Methods:**

Brachial artery FMD and GMD and carotid intima media thickness (cIMT) were studied using ultrasound in 20 patients diagnosed with early RA in whom symptoms had been present for less than 12 months, and in 20 control subjects matched for age, sex and established cardiovascular risk factors. FMD and GMD were re-assessed after 12 months in RA patients and the change in each parameter was calculated. Data were analysed by univariate regression.

**Results:**

Mean FMD and GMD were significantly lower in early RA patients at baseline than in controls, but each parameter significantly improved in one year. FMD and GMD responses were positively associated with each other. Patients' age, C-reactive protein (CRP) level and cIMT at baseline and CRP level at one year, were negatively associated with change in brachial responses in one year.

**Conclusions:**

Patients with recent-onset RA have altered brachial artery responses signifying both functional and structural abnormalities. However, early control of inflammation may reduce arterial dysfunction and thus the tendency for atherosclerotic progression.

## Introduction

Patients with rheumatoid arthritis (RA) experience cardiovascular (CV) events more often than expected [1] and their mortality attributable to CV causes is increased [2]. Furthermore, ischaemic heart disease (IHD) has been found to occur about 10 years earlier in patients with RA compared with a population of patients with osteoarthritis matched for classical CV risk factors. Indeed, it has been suggested that RA itself is an independent risk factor for IHD [3]. The similarities which exist between the inflammatory/immunological reaction in RA and atherosclerosis raise the possibility that inflammatory mechanisms responsible for synovial lesions might also involve the vessel wall to facilitate the development of atherosclerotic lesions [4].

Although atherosclerosis can manifest as overt CV disease, it can be detected at an earlier stage by recognition of abnormal endothelial function and elevated carotid intima media thickness (cIMT) as measured by ultrasound. These measures correlate closely with direct measurement of local and systemic atherosclerotic burden in studies of pathology and with clinical CV endpoints [5,6]. Ultrasonographic assessment of the common carotid artery is a feasible, reliable, valid and cost-effective method for both population studies and clinical trials of atherosclerosis progression and regression [7]. We showed that cIMT is significantly higher in patients presenting with early RA than in controls matched for age, sex and CV risk factors [8].

Endothelial function can be assessed by ultrasound as flow-mediated vasodilatation of the brachial artery in response to increased vessel wall shear stress and mediated by nitric oxide (NO) release by endothelial cells [9,10]. Brachial artery vasodilatation can also be assessed by exogenous NO after sublingual glyceryl trinitrate (GTN) [11]. Post-GTN vasodilatation is considered endothelium-independent and predominantly mediated by smooth muscle. The dose-response curve for GTN-mediated vasodilatation has been examined in subjects with proven coronary artery disease and in healthy controls [12]. Patients with coronary disease had significantly reduced GTN-mediated vasodilatation, with the greatest difference observed with lower doses of GTN, suggesting that atherosclerosis is associated with functional abnormalities of both endothelium and vascular smooth muscle cells [12].

A previous study of 10 patients with early RA demonstrated impaired flow-mediated endothelial function, with improvement over six months of therapy [13]. Moreover, infliximab therapy improved endothelial-dependent vasodilatation, apparently through direct endothelial, rather than systemic effects [14]. In women without RA, treatment-associated improvement in endothelium-dependent vasodilatation has been shown to decrease the risk of subsequent CV events [15]. In longstanding RA, endothelial dysfunction is predicted by the C-reactive protein (CRP) level [15-19]. Other acute and chronic inflammatory states have also been shown to impair endothelial function [20-22]. In the current study, we studied a group of early RA patients, in whom cIMT had also been determined, and investigated the progression of endothelium-dependent and -independent brachial arterial function after one year.

Given the relationship of inflammation severity to atherosclerotic burden, as measured by cIMT and carotid plaque, at presentation with early RA [23], we hypothesised that inflammatory indices would predict arterial responses. Therefore, a group of conventional CV risk factors and indices of RA inflammatory disease were analysed at baseline and one year to determine their relationship to changes in brachial artery response.

## Materials and methods

### RA patients

All 31 study participants met the American College of Rheumatology 1987 revised criteria for the classification of RA [24]. All the patients were enrolled from 2004 to 2005 with a diagnosis of RA with symptom duration less than 12 months. Patients attended an early RA clinic regularly, and received combination methotrexate (MTX), sulfasalazine (SSZ) and hydroxychloroquine (HCQ) [25], unless contraindicated, after diagnosis and active disease were confirmed. After diagnosis, patients were treated according to a response-driven step-up algorithm, as previously described [26], with the aim of achieving clinical remission [27-29]. Intra-articular but not oral corticosteroids were used to control inflammation in addition to disease-modifying antirheumatic drugs (DMARDs), as required.

At one year, 52% were taking MTX, SSZ and HCQ, 6% MTX and SSZ, 16% MTX and HCQ, 10% SSZ and HCQ and 6% SSZ only, and 10% refused treatment. An additional 20 healthy subjects were recruited from the community, who could be matched for age, sex and CV risk factors against 20 of the early RA subjects. We matched against a database of more than 1000 individuals recruited in a primary prevention setting. The primary match was with the age and gender, after which we matched on number and type of risk factors on a categorical basis. Although exact blood pressure or lipid levels were not matched, we were able to match for hypertension and hyperlipidaemia, in addition to smoking status in almost all the cases.

### Study procedure

The study was approved by the human research ethics committee at Princess Alexandra Hospital, Woolloongabba, and all subjects provided written informed consent. CV risk factors were ascertained among RA patients at baseline, as previously described [8]. RA disease activity parameters and laboratory measurements were assessed as previously described, at baseline and 12 months. Flow-mediated dilatation (FMD), GTN-mediated dilatation (GMD), cIMT and plaque were measured soon after the diagnosis of RA was confirmed; FMD and GMD were repeated after 12 months and as described [8].

### Vascular function testing

Brachial artery flow and GMD testing was undertaken within one to four weeks after diagnosis and before commencement of DMARDs. Individual blood pressure and heart rate remained constant during the testing. No subject had ultrasound evidence of brachial artery atherosclerotic plaque. Ultrasonography was performed by a trained investigator (SH) who was unaware of the subject's clinical data, using a high-resolution ultrasound machine, according to the International Brachial Artery Reactivity Task force Guidelines [30]. Two patients were taking statins, one was taking an angiotensin-converting enzyme inhibitor and one patient was taking both. Although all four patients were taking a once daily dose of their medication, they were asked to postpone this medication until after the vascular ultrasound. No patient was taking aspirin or GTN treatment.

Subjects rested in a supine position in a quiet, dark, temperature-controlled room. A pneumatic cuff was placed around the upper forearm distal to the segment of brachial artery, scanned in longitudinal section 2 to 10 cm above the antecubital crease. Focus, depth and gain were individually set to optimise images of the lumen/arterial wall interface. A baseline scan was recorded for two minutes, followed by induction of hyperaemia by cuff inflation to 240 mmHg for four minutes. The FMD scan commenced 30 seconds before release of the cuff, and continued for one minute afterwards. A second baseline scan was recorded 10 minutes later. The coefficient of variation (cv) was 0.18 for FMD. A tablet of GTN was administered sublingually in a standardised manner and recording was continued for a further four minutes for GMD. The cv was 0.17 for GMD. Measurement of the brachial artery diameter was synchronised with the R wave of the electrocardiogram, to avoid possible errors resulting from artery pulsation. FMD and GMD were expressed as the relative increase in brachial artery diameter during hyperaemia:



cIMT was measured using carotid duplex scanning and automated software as previously described [31]. The cv for cIMT was 0.2.

### Statistical analysis

In a preliminary evaluation, continuous variables were tested for normality of distribution; transformations were applied for non-normally distributed variables. Variables with normal distribution were expressed as the mean ± standard deviation (SD) and categorical variables as percentages. Differences between the FMD and GMD in RA and control groups were compared using the two-sample (independent) Student's t-test for normally distributed data. Log transformations were applied to non-normally distributed data. Associations of FMD and GMD with age, continuous CV variables and disease activity variables were evaluated using linear regression analysis. Brachial reactivity improvement, inflammatory and RA disease activity markers and lipid profile were summarised using mean values for the baseline measures and compared with the mean values at one year using paired Student's t-tests. The differences between the mean values were examined by two-sample Student's t-tests. Linear regression analysis examined univariate correlations, and two-sided values of *P *< 0.05 were regarded as statistically significant. For all the analyses, Stata 9/SE (Stata Corp, College Station, TX, USA) statistical software was used [8].

## Results

### Clinical features

Thirty-one patients presenting with RA within 12 months of symptom onset (Table 1) were treated and followed clinically for one year. Of the patients, 90% were Caucasian, 3% were Australian aboriginal and 6% Asian. Four patients had previous CV events, including one male with a previous transient ischaemic attack at 52 years of age, two males with a history of angina at the ages of 48 and 64 years, and one patient with myocardial infarction (MI) at age 48 years. Twenty of these RA patients could be matched for age, sex and CV risk factors against 20 healthy control subjects from a database of more than 1000 individuals recruited in a primary prevention setting.

**Table 1:** Patient details at baseline and one year

	**Week 0** (mean ± SD)	**Week 52**	***P *value**
**Demographic details**			
Male:female	19:12	**-**	**-**
Mean age (range, years)	54 (23 to 78)	**-**	**-**
Body mass index (range, kg/m^2^)	28 (17 to 45)	**-**	**-**
**Rheumatoid arthritis characteristics**			
Disease duration, mean (range, months)	1.8 (1 to 12)	**-**	**-**
Tender joint count (out of 53)	18 (14)	7.6 (8.1)	**< 0.001**
Swollen joint count (out of 44)	15 (8.9)	5.0 (9.3)	**< 0.001**
Health assessment questionnaire score (maximum disability 24)	3.3 (3.5)	1.8 (2.7)	**0.006**
Physician's global assessment of disease activity (maximum 100)	37 (27)	26 (28)	**0.013**
Joint pain (VAS, maximum 100)	51 (32)	24 (26)	**< 0.001**
Rheumatoid factor level	281 (438)	98 (127)	**0.011**
Disease activity score, 4 v	4.3 (1.6)	2.6 (1.4)	**< 0.001**
Rheumatoid factor positive, n (%)	21 (68%)	17 (55%)	
**Cardiovascular risk factors**			
History of ever smoking, n (%)	20 (65%)	**-**	**-**
History of current smoking, n (%)	8 (26%)	**-**	**-**
History of hypertension, n (%)	6 (19%)	**-**	**-**
History of diabetes mellitus, n (%)	3 (10%)	**-**	**-**
History of hyperlipidaemia, n (%)	2 (6.5%)	**-**	**-**
History of myocardial infarction, n (%)	1 (3.2%)	**-**	**-**
History of angina, n (%)	2 (6.5%)	**-**	**-**
History of stroke and/or transient ischaemic attack, n (%)	1 (3.2%)	**-**	**-**
Family history of cardiovascular disease, n (%)	6 (19%)	**-**	**-**
**Laboratory values**			
Erythrocyte sedimentation rate (mm/hour)	40 (25)	21 (16)	**< 0.001**
C-reactive protein (mg/L, normal < 6)	27 (25)	9.3 (12)	**< 0.001**

Bold values are statistically significant. SD = standard deviation; VAS = visual analogue scale.

### Baseline cIMT, FMD and GMD in 20 patients with early RA and 20 matched controls

**Figure 1:**
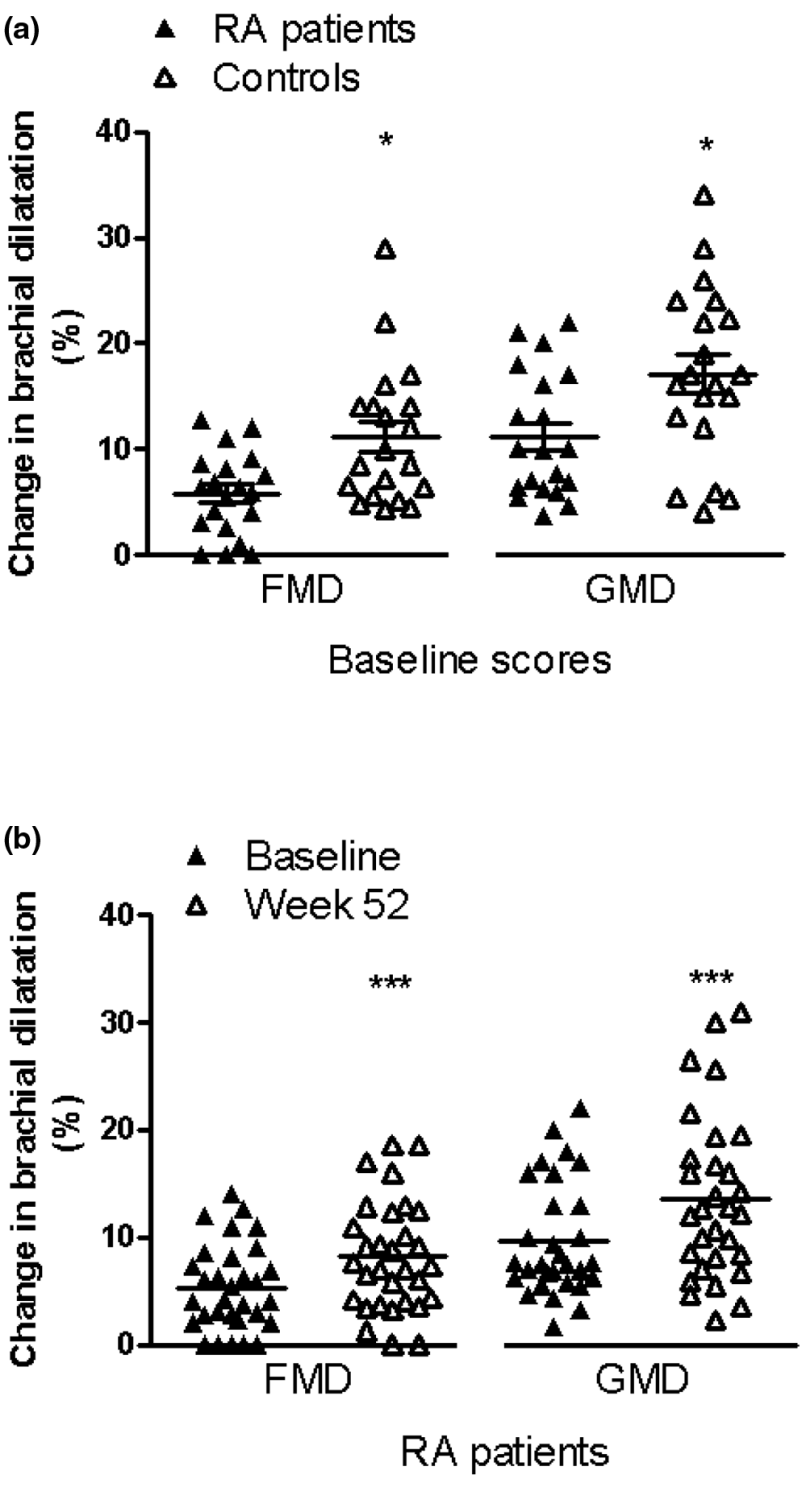
Brachial dilatation of 20 patients with early RA and matched controls, and of 31 patients with early RA at baseline and one year. Flow-mediated and GTN-mediated dilatation of the brachial artery was measured using ultrasound as described in the methods. Results presented as mean ± standard deviation. Flow mediated vasodilatation, 100 × (post-hyperaemic diameter-basal diameter)/basal diameter, GTN-mediated dilatation, 100 × (post-GTN diameter-basal diameter)/basal diameter. **(a) **RA patients are compared with healthy controls matched for age, sex and CV risk factors. **(b) **RA patients are compared at baseline and after one year of treatment with anti-rheumatic drugs. FMD = flow-mediated dilatation; GMD = GTN-mediated dilatation; GTN = glyceryl trinitrate; RA = rheumatoid arthritis.

The mean age at the time of diagnosis was 45 years (range 23 to 64 years), with male patients significantly older than females (51.2 ± 9.2 years vs. 40.2 ± 10.7 years, *P *= 0.03). The mean duration of RA symptoms at the time of scanning was 6 ± 3 months (range 1 to 12 months). Mean FMD and GMD of the brachial artery were significantly lower in RA patients than in controls (Figure 1a). We repeated the analysis after removing patients with previous CV events and their matched controls, and the results did not differ (FMD 5 ± 4 vs. 11 ± 7, *P *= 0.001 and GMD 11 ± 6 mm vs. 17 ± 8 mm, *P *= 0.02).

### FMD and GMD annual change and determinants of progression

The annual changes in FMD and GMD measured by ultrasound were determined. FMD and GMD improved significantly over one year (Figure 1b). Univariate analyses were carried out to determine CV risk factors or inflammatory factors which might contribute to these changes in vascular parameters in patients with early RA over the first year. Improvements in FMD and GMD over one year were correlated. Age, baseline CRP and baseline cIMT were negatively correlated with the change in both FMD and GMD (Table 2). CRP level at one year was also negatively associated with the change in FMD and GMD. Patients who presented with high CRP levels at RA disease onset were more likely to continue with high CRP levels by one year (data not shown, *P *< 0.03). These data suggest that endothelial dysfunction is reversible with falling levels of CRP. In support of this idea, erythrocyte sedimentation rate (ESR) level at baseline exhibited a similar negative relationship with FMD progression, and rheumatoid factor (RF) positive patients were less likely to improve FMD (Table 2). There were no significant associations of FMD with health assessment questionnaire (HAQ), gender, smoking history, blood pressure or lipid levels. In contrast, history of smoking ever and smoking pack year history were negatively correlated with change in GMD, consistent with the utility of GMD as a structural measure of vascular disease (Table 2). There were no significant associations of GMD with HAQ, ESR, blood pressure, high-density lipoprotein or triglyceride levels.

**Table 2:** Univariate analysis of the relationship between change in flow-mediated dilatation and GTN-mediated dilatation, features of RA and CV risk factors in 31 patients with early RA

	**R^2^**	**β coeff**	P
**FMD:**			
RF presence	-	-	**0.037**
**Log CRP level**			
At week 0	0.173	-0.946	**0.020**
At week 52	0.319	-1.700	**0.001**
**ESR**			
At week 0	0.142	-1.451	**0.037**
At week 52	0.001	-0.065	0.91
**Change in GMD**	0.428	0.487	**< 0.001**
**Carotid IMT at baseline**	0.196	-10.22	**0.013**
**Age**	0.346	-0.133	**< 0.001**
**Post menopausal status**	-	-	**0.002**
**GMD:**			
**Log CRP level**			
At week 0	0.214	-0.394	**0.006**
At week 52	0.431	-0.693	**< 0.001**
**Change in FMD**	0.428	0.880	**< 0.001**
**Carotid IMT at baseline**	0.232	-3.89	**0.007**
**Age**	0.206	-0.035	**0.012**
**Pack year history**	0.173	-0.148	**0.022**
**Ever smoking**	-	-	**0.02**
**LDL**			
At week 0	0.173	-0.561	0.086
At week 52	0.352	-0.552	**0.015**

Parameters showing statistically significant relationships are included. Bold values are statistically significant.

CRP = C-reactive protein; CV = cardiovascular; ESR = erythrocyte sedimentation rate; FMD = flow-mediated dilatation; GMD = GTN-mediated dilatation; GTN = glyceryl trinitrate; IMT = intima-media thickening; LDL = low-density lipoprotein; RA = rheumatoid arthritis; RF = rheumatoid factor.

## Discussion

An increasing body of evidence indicates that atherosclerosis shares similarities with other inflammatory/autoimmune diseases; indeed, there are surprising similarities between the inflammatory response observed in atherosclerosis and RA [32-34]. The current study hypothesised that the severity of systemic inflammation would be associated with progression of arterial dysfunction in RA. Patients with RA are at significantly higher risk of death than an age-matched population [35] with CV disease as a major cause of increased mortality [1], and discovery of early interventions in RA disease that might influence atherosclerotic progression are essential to determine approaches that could in turn reduce the subsequent risk of CV mortality. Of importance, the current studies clearly demonstrate the effect of early DMARD intervention, and the effect of reduction in CRP on vascular endothelial dysfunction and GTN-mediated brachial arterial function during the first year of RA. Furthermore, while each vascular measurement indicates a different aspect of vascular dysfunction – FMD is a measure of endothelial function [9,10] and GMD of smooth muscle damage [12] – each was reversible with treatment, and annual changes in each measure were correlated.

Arterial compliance is contributed by the arterial media layer, with abnormalities resulting from combined endothelial and smooth muscle damage [36]. Reduction in CRP in patients with RA was associated with substantial and significant improvement in both endothelium-dependent and endothelium-independent skin microvascular dysfunction [37,38]. A similar improvement in FMD and GMD occurred after treatment of early RA patients in the present study.

Disease activity and functional impairment are predictive of mortality in RA. Thus, if effective therapy could be introduced prior to the development of arterial damage, outcome could be improved. It has been demonstrated extensively that early therapeutic intervention with anti-rheumatic drugs improves the prognosis of RA [39], and that disease duration is a significant determinant of response to therapy [40]. The strategy of combined DMARDs in early RA shows a beneficial effect [41,42]. We previously demonstrated that patients with early RA had significantly higher average cIMT values and more plaque than healthy controls matched for CV risk factors. cIMT was predicted by age and CRP level at first presentation with RA. A recent small study demonstrated that baseline cIMT in patients with established RA predicted development of CV events over the next five years [43]. Our results suggest that baseline CRP, ESR and RF may be useful prognostic markers for CV disease along with non-invasive measures of vascular function, including cIMT, FMD and GMD.

The lack of improvement in traditional CV risk factors in RA patients despite the improvement in FMD and GMD, which were both positively correlated with improvement in CRP, further implicates inflammation in arterial function impairment. Moreover, it is likely that endothelial function in RA primarily depends on the control of inflammatory activity rather than the specific treatment, as demonstrated by improvement with a variety of effective anti-rheumatic agents [16,44-46]. MTX was shown to reduce overall mortality by 60% primarily by reducing mortality from coronary heart disease [47]. The mechanism by which MTX provides CV protection might be explained at least in part by suppression of systemic inflammation. Although endothelial dysfunction has been reported in RA patients treated with long-term MTX [48], the improvement of endothelial function in our patients might be explained by early suppression of inflammation compared with the treatment of established RA patients with chronic inflammatory effects on vessels. In another study, reversal of endothelial dysfunction in patients with long-standing severe RA was only transient in response to TNF inhibitors, despite good control of systemic inflammation, suggesting that structural vascular changes might preclude more prolonged effects [45]. A recent demonstration that abnormal FMD was associated with longer disease duration in patients with established RA also supports the concept that endothelial dysfunction progresses and becomes less reversible over time [49].

Traditional CV risk factors identified from epidemiological studies such as hyperlipidaemia, smoking and older age may interact to damage the endothelium and smooth muscle in asymptomatic patients in the same way as they are known to interact to determine the risk of clinical CV endpoints [50]. Our data demonstrate that improvement in GMD, a marker of endothelial and smooth muscle dysfunction, after one year of treatment of early RA was significantly negatively associated with pack year history of smoking. Cigarette smoking increases oxidative stress because of low circulating levels of antioxidants and increased levels of oxygen-derived free radicals and lipid peroxides that degrade NO, leading to endothelial dysfunction [51,52]. Thus, smoking history may have a specific impact on the capacity of the endothelium and smooth muscle to respond to the anti-inflammatory effects of treatment. In a study of patients with longstanding RA, where those with a history of CV disease or CV risk factors including smoking were excluded, GMD in the RA patients was no different to that of age- and sex-matched controls or of the healthy control subjects in the current study [49]. These data suggest the hypothesis that inflammatory arthritis in the absence of CV risk factors does not promote arterial smooth muscle damage.

## Conclusions

This study shows that inflammation severity is closely associated with functional and structural arterial wall changes in patients with early RA. Early control of inflammation is associated with improved arterial function which may reduce atherosclerotic progression.

## Abbreviations

cIMT: carotid intima-media thickening; CRP: C-reactive protein; CV: cardiovascular; DMARDs: disease-modifying anti-rheumatic drugs; ESR: erythrocyte sedimentation rate; FMD: flow mediated dilatation; GMD: GTN-mediated dilatation; GTN: glyceryl trinitrate; HAQ: health assessment questionnaire; HCQ: hydroxychloroquine; IHD: ischaemic heart disease; MTX: methotrexate; NO: nitric oxide; RA: rheumatoid arthritis; RF: rheumatoid factor; SD: standard deviation; SSZ: sulfasalazine; TNF: tumour necrosis factor.

## Competing interests

The authors declare that they have no competing interests.

## Authors' contributions

SH, TM and RT were involved in conception, design, acquisition, analysis, interpretation of data and drafting and revising the manuscript: All authors read and approved the final manuscript.
